# Integrative Analysis of Cytokine and Lipidomics Datasets Following Mild Traumatic Brain Injury in Rats

**DOI:** 10.3390/metabo14030133

**Published:** 2024-02-21

**Authors:** Alexis N. Pulliam, Alyssa F. Pybus, David A. Gaul, Samuel G. Moore, Levi B. Wood, Facundo M. Fernández, Michelle C. LaPlaca

**Affiliations:** 1 Coulter Department of Biomedical Engineering, Georgia Institute of Technology/Emory University, Atlanta, GA 30332, USAafpybus@gatech.edu (A.F.P.); levi.wood@me.gatech.edu (L.B.W.); 2 Petit Institute for Bioengineering and Bioscience, Georgia Institute of Technology, Atlanta, GA 30332, USA; 3 School of Chemistry and Biochemistry, Georgia Institute of Technology, Atlanta, GA 30332, USA; 4 George W. Woodruff School of Mechanical Engineering, Georgia Institute of Technology, Atlanta, GA 30332, USA

**Keywords:** lipidomics, traumatic brain injury, neuroinflammation, cytokines, multi-omics

## Abstract

Traumatic brain injury (TBI) is a significant source of disability in the United States and around the world and may lead to long-lasting cognitive deficits and a decreased quality of life for patients across injury severities. Following the primary injury phase, TBI is characterized by complex secondary cascades that involve altered homeostasis and metabolism, faulty signaling, neuroinflammation, and lipid dysfunction. The objectives of the present study were to (1) assess potential correlations between lipidome and cytokine changes after closed-head mild TBI (mTBI), and (2) examine the reproducibility of our acute lipidomic profiles following TBI. Cortices from 54 Sprague Dawley male and female rats were analyzed by ultra-high-performance liquid chromatography mass spectrometry (LC-MS) in both positive and negative ionization modes and multiplex cytokine analysis after single (smTBI) or repetitive (rmTBI) closed-head impacts, or sham conditions. Tissue age was a variable, given that two cohorts (*n* = 26 and *n* = 28) were initially run a year-and-a-half apart, creating inter-batch variations. We annotated the lipidome datasets using an in-house data dictionary based on exact masses of precursor and fragment ions and removed features with statistically significant differences between sham control batches. Our results indicate that lipids with high-fold change between injury groups moderately correlate with the cytokines eotaxin, IP-10, and TNF-α. Additionally, we show a significant decrease in the pro-inflammatory markers IL-1β and IP-10, TNF-α, and RANTES in the rmTBI samples relative to the sham control. We discuss the major challenges in correlating high dimensional lipidomic data with functional cytokine profiles and the implications for understanding the biological significance of two related but disparate analysis modes in the study of TBI, an inherently heterogeneous neurological disorder.

## 1. Introduction

Traumatic brain injury (TBI) is a significant source of disability, with a global annual incidence of 69 million [1], and may lead to long-lasting neurological deficits and a decreased quality of life for patients across injury severities. Approximately 80% of TBIs are considered mild [2]; mild TBI (mTBI) has been known as the silent epidemic because of its load on acute healthcare [3,4]. Following the primary injury phase, TBI is characterized by complex secondary cascades that include oxidative stress [5], excitotoxicity [6], blood–brain barrier (BBB) damage [7], plasma membrane damage [8], cell death [9], neuroinflammation [10], and lipid dysregulation [11] in the acute, subacute, and chronic phases after injury. In this study, we focused on lipid dysregulation and neuroinflammation changes after mTBI, given the relationships between lipids and inflammation.

Lipidomics is relatively underexplored in preclinical and clinical TBI studies [11,12]. Recent advances in mass spectrometry have facilitated a high volume of data output; however, it remains a challenge to distinguish normal cellular turnover from pathological cascades. Lipids play important roles in cellular function, including energy production, cell structure, and signaling pathways. Lipids also mediate pathophysiological processes and inflammation [13]. Lipid dysregulation after TBI from oxidative stress, mitochondrial dysfunction, and excitotoxicity may lead to the degradation of and damage to neuronal and glial membranes [12,14]. For example, following a moderate TBI, phospholipase A_2_ (PLA_2_) activity increases as early as within 15 min [15], leading to the release of downstream free fatty acids and oxidized lipids. Investigating lipidome changes after TBI may lead to the identification of biomarkers and therapeutic targets.

Neuroinflammation in the acute and subacute periods following a TBI is well established in preclinical and clinical reports [10,16] and elevated proinflammatory cytokine profiles have been linked to poor health outcomes in moderate to severe TBIs [17,18]. For example, neuroinflammation involves changes at the level of genes, metabolites, lipids, and proteins, that vary depending on brain location and variables such as time of day and stress state [18]. Cytokines are pleiotropic, producing multiple different and overlapping effects on various cell types [19] and can have both anti- and pro-inflammatory effects [20]. Although cytokine and lipids are involved in similar signaling pathways, there has been minimal exploration of linking lipids to neuroinflammation changes after TBI [11].

Despite decades of research investigating the pathophysiology of TBIs and testing of mechanistic-based neurotherapeutics, there are no United States Food and Drug Administration (FDA)-approved drugs that directly target neuroprotection [21]. The advances in multi-plexed and multi-omics approaches open opportunities for the discovery of novel targets and combination therapies. Finding correlations among disease-related changes and omics in terms of tens of thousands of genes, proteins, and lipids, has the potential to advance our understanding of complex interactions, generating layers of information. With the opportunity of multi-dimensional analyses, there are many challenges, including the identification of molecules, determining meaningful interactions, managing the absence of key molecules and unknown systemic influences, and temporal alignment of relevant molecular events. A previous study from our lab compared changes in the brain and serum after mTBI where there were different temporal profiles between each compartment 24 h post TBI [22]. The lipids decreased in the cortices and increased in the serum relative to sham control, possibly due to an examination of a single time point and missing information. Despite these challenges, exploring multi-omic approaches and surveying multisource tissue or biofluid has tremendous potential for furthering our understanding of biochemical injury cascades after a TBI.

In the present study, we compared lipidomic and cytokine changes in cortical brain tissue from male and female rats 24 h following single and repetitive mTBI in a clinically relevant closed-head-injury model [23]. The goals were to test batch effects in two cohorts and to correlate lipidomic and cytokine changes due to TBI. We also discuss the major challenges in correlating high dimensional lipidomic data with cytokine expression and the implications for understanding the biological significance of two related but disparate analysis modes in the study of TBI, which is an inherently heterogeneous neurological disorder.

## 2. Material and Methods

### 2.1. Injury Protocol

All animal procedures were performed in accordance with guidelines set forth in the Guide for the Care and Use of Laboratory Animals (U.S. Department of Health and Human Services, Washington, DC, USA, Pub no. 85-23, 1985) and were approved by the Georgia Institute of Technology Institutional Animal Care and Use Committee (protocol #A100188). Experimental batch 1 and 2 Sprague Dawley rats (obtained at 8 weeks old; Charles River, Wilmington, MA, USA) weighed 400 g on average on injury day and were kept on 12 h reverse light–dark cycles, with food and water available ad libitum. Animals were randomly assigned by a random generator (https://www.random.org/lists/ accessed in 1 July 2019) to either sham, single impact, or repetitive impacts. Experimental batch 1 contained female (*n* = 16) and male (*n* = 10) assigned to either sham procedure (*n* = 10), 1X, smTBI (*n* = 8), or 3X, rmTBI (*n* = 8) groups. Experimental batch 2 contained female (*n* = 11) and male (*n* = 17) assigned to either sham procedure (*n* = 8), single impact (*n* = 9), or repetitive impact (*n* = 11) groups.

A modified, controlled cortical impact (CCI) device (Pittsburgh Precision Instruments, Pittsburgh, PA, USA) with a 1 cm diameter silicone stopper (Renovators Supply Manufacturing, Erving, MA, USA) added to the standard CCI piston was used to induce single and repetitive closed-head impacts. Rats were anesthetized with isoflurane (induction: 5% isoflurane; maintenance: 3% isoflurane) and removed from anesthesia 30 s prior to closed-head impacts, similar to previously reported studies [24,25]. It has been suggested that anesthesia artifacts [26,27,28,29,30] compromise preclinical TBI model fidelity [31], so we allowed partial recovery from anesthesia prior to impact balances not using anesthesia [32,33] with the known neurological confounds of commonly used anesthetics. Rats were placed in prone position on 1-inch-thick ethylene-vinyl acetate foam (McMaster-Carr, Elmhurst, IL, USA). The impacts were delivered at the midpoint between the bregma and lambda skull suture landmarks on the dorsal surface of the closed head. All mTBI groups received impacts from the piston at a velocity of 5 m/s. The single impact (smTBI, 1X) group received one impact with a 5 mm head displacement. The repeat impact (rmTBI, 3X) group received a total of 3 impacts with 2 min intervals between impacts with head displacements 5 mm, 2 mm, and 2 mm. It is worth noting that the majority of repetitive closed head impact models use 24 h intervals, with ranges from 3 min to 1 month [34,35]. Repetitive impacts occurring in the order of minutes is intended to model real-world situations like military and sports-related repetitive impacts [34,36]. Sham animals received procedures identical to injured animals, excluding impacts. Righting latency was recorded as an acute neurological indicator of injury following the last impact. The righting reflex is a measure of recovery from unconsciousness that in analogous to loss of consciousness in humans [37,38] and has been shown to correlate with behavioral and histopathological measures of injury severity in animal models of TBI [39,40].

### 2.2. Sample Collection and Preparation

Brain samples were harvested and collected following transcardial perfusion with phosphate buffer (0.1 M, pH 7.4) 24 h post TBI. The perfused whole brains were rapidly removed, and flash frozen in an isopentane–methanol ice slurry. Pieces of parietal cortices (5 mm × 2 mm) were dissected from partially thawed brains by removing the subcortical structures including the majority of white matter and stored at −80° C in microcentrifuge tubes. The cortices were then transferred to liquid nitrogen and manually pulverized with a pestle and mortar submerged in liquid nitrogen and aliquoted in ~10–30 mg tissue samples, that were used for both LC-MS and cytokine analyses.

The experiments on the experimental batch 1 animals were completed 1.5 years before experimental batch 2. Experimental batch 1 and 2 aliquoted tissue samples were thawed simultaneously on ice prior to addition of solvent (IPA and Splash II Lipidomix in (1:3 *v/v*)) to separate lipids and small non-polar metabolites. LC-MS grade water was used to prepare sample blanks, and pooled quality control (QC) samples were prepared from 5 µL aliquoted supernatant of all samples in the study. The brain, solvent (1:4 *w*/*v*), and beads were placed in a Tissuelyser II for 8 min and centrifuged at 16,000× *g* for 7 min. The supernatant was collected for LC-MS. Pooled quality control samples were formed by combining 6 µL aliquots of all brain sample extracts. Sample blanks were prepared with the same procedure, except instead of a brain sample, 50 µL of LC-MS grade water was used. Both experimental batches 1 and 2 underwent 1 freeze–thaw cycle between brain harvest and LC-MS analysis, thus the main difference between batches was duration of storage.

### 2.3. Sample Analysis with Ultra-High Performance Liquid Chromatography–Mass Spectrometry (UPLC-MS)

Samples were analyzed using a Vanquish Horizon UHPLC instrument coupled to an ID-X Orbitrap Tribrid mass spectrometer operated in both positive and negative ion modes. Both ion modes used the identical two-part mobile phase gradient. Mobile phase A was a (40:60 *v*/*v*) water/ACN mixture and mobile phase B was a (90:10 *v*/*v*) IPA/ACN mixture. Mobile phases A and B each contained 0.1% formic acid and 10 mM ammonium formate. The stationary phase used for both ionization modes was a 2.1 mm × 50 mm Accucore C30 column with 2.1 µm particle size. Samples were randomized and analyzed over a range of 150–2000 *m/z*. Detailed UPLC-MS methods were previously described [22,41].

LC-MS/MS experiments were conducted using a data-dependent acquisition (DDA) strategy to aid in compound identification. MS spectra were collected with a resolution of 30,000 and the dd-MS2 were collected at a resolution of 15,000 and an isolation window of 0.8 *m/z*. Precursors were fragmented with higher energy collisional dissociation (HCD) and collision-induced dissociation (CID) activation. Stepped normalized collision HCD energies of 15%, 30%, and 45% fragmented selected precursors in the collision cell and produced ions were detected in the orbitrap. Normalized CID energy 45% fragmented and analyzed ions in the ion trap. Dynamic exclusion was set at 6 s and duty cycle was set to 1 s.

### 2.4. UPLC-MS Data Processing

Raw LC-MS data were processed using Compound Discoverer v3.0.0 software and the XCMS web-based application and used to identify internal standards. Initial processing steps include retention time peak alignment between samples, peak detection, peak area integration, isotope peak grouping, adduct peak grouping, gap filling, and drift correction. Features eluting with the solvent front or having retention times below 0.75 min were removed from the dataset to account for potential ion suppression effects.

### 2.5. Luminex Methods

Cryopulverized cortices (Methods 2.2) were lysed using the Bio-Plex cell lysis kit (Bio-Rad Laboratories #171304011, Hercules, CA, USA) and protein concentrations were determined using a Pierce BCA Protein Assay (Thermo Fisher #23225, Waltham, MA, USA). Multiplexed cytokine quantification was conducted using the Milliplex^®^ MAP Rat Cytokine/Chemokine Magnetic Bead Panel—Immunology Multiplex Assay for the following 26 cytokines: EGF/epidermal growth factor*, eotaxin/C-C motif chemokine 11, CCL11, Fractalkine/CX3CL1, G-CSF/granulocyte colony stimulating factor*, GM-CSF/granulocyte-macrophage colony stimulating factor*, GRO/KC/growth-regulated oncogene/keratinocyte chemoattractant*, IFN-γ/interferon gamma*, IL-1α/interleukin 1-alpha, IL-1β/interleukin 1-beta, IL-2/interleukin-2*, IL-4/interleukin-4*, IL-5/interleukin-5, IL-6/interleukin-6, IL-10/interleukin-10*, IL-12 (p70)/interleukin-12p70*, IL-13/interleukin-13, IL-17A/interleukin-17A, IL-18/interleukin-18, IP-10/interferon γ-induced protein 10 kDa (C–X–C motif chemokine 10, CXCL10), Lepton*, LIX/lipopolysaccharide-induced CXC chemokine, MCP-1*, MIP-1α*, MIP-2/monocyte chemoattractant protein-1, RANTES/Regulated upon Activation, Normal T cell Expressed, and Secreted (C–C chemokine ligand 5, CCL5), TNF-α/tumor necrosis factor-alpha, VEGF/vascular endothelial growth factor) (Millipore Sigma RECYTMAG-65K. The Luminex cytokine panel was selected because we and others have found that these cytokines have both pro- and anti-inflammatory properties and have been shown to change following TBI [10,18,42]. Cytokines marked with an asterisk did not fall within a linear range and were not included in our analysis (leaving 14 cytokines). Prior to analysis, lysates were thawed on ice and centrifuged at 4 °C for 10 min at 15,500× *g*. Protein concentrations were normalized with Milliplex^®^ MAP Assay Buffer (EMD Millipore, Billerica, MA, USA) to 4 μg protein per 37.5 μL assay volume. This protein concentration was selected because it fell within the linear range of bead fluorescent intensity versus protein concentration for 15 detectable analytes. All kits were read on a MAGPIX^®^ system (Luminex, Austin, TX, USA).

### 2.6. Statistical Analysis

An ANOVA was calculated by Compound Discoverer on logarithmic transformed data with Tukey HSD post hoc for total LC-MS dataset. An unpaired *t*-test with Welch’s correction was used to compare sham batch 1 and sham batch 2 groups to remove features that were statistically different between batches on logarithmic transformed data. Mixed effect model was used to calculate the effects of injury on cytokine profiles. Data were analyzed using GraphPad Prism 8. Reported *p* values are multiplicity adjusted to account for multiple comparisons. For all cases, significance was defined as *p* < 0.05 (*) or *p* < 0.01 (**), *p* < 0.001 (***), or *p* < 0.0001 (****).

## 3. Experimental Design

**Figure 1:**
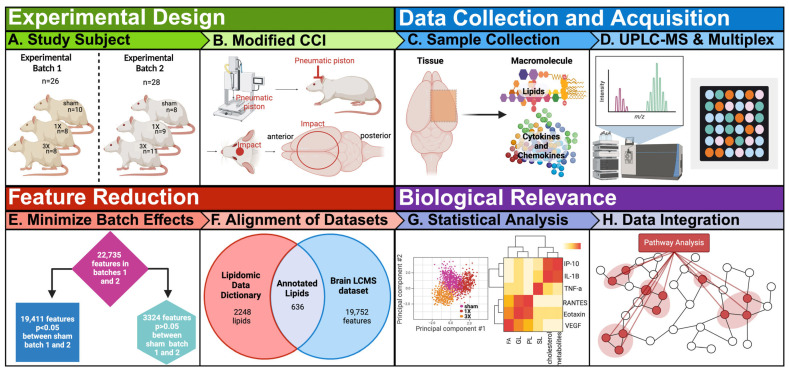
Overview of study design, data acquisition, feature reduction, and biological relevance. (**A**) Rats were randomly assigned to different injury groups. Experimental batch 1 group were sham control group that received no injuries (*n* = 10), single impact group (smTBI) that received one impact (1X, *n* = 8), and repeat impact group (rmTBI) that received three separate impacts (3X, *n* = 8) with both males (*n* = 10) and females (*n* = 16). Experimental batch 2 group included sham control group that received no injuries (*n* = 8), smTBI group that received one impact (*n* = 9), and rmTBI group that received three separate impacts (*n* = 11) with both males (*n* = 17) and females (*n* = 11). (**B**) The rats received closed head impacts with a pneumatic driven controlled cortical impact (CCI), modified for closed head injury. (**C**) Brains were collected at 24 h post-injury and cryopulverized in preparation for lipid and cytokine analysis. (**D**) Samples were analyzed with Luminex multiplex assay to obtain cytokine panels and high-resolution LC-MS. Spectral alignment, peak detection and isotope and adduct grouping, gap filling, and drift correction were accomplished using Compound Discoverer v.3.0 to obtain the lipidomic dataset. (**E**) The features were reduced by Welch’s *t*-test to minimize batch effects in the lipidomic dataset. (**F**) The lipidomic dataset was annotated with the in-house database based on exact mass, retention time, and spectral matching. (**G**) Annotated lipids and cytokine panel were analyzed with volcano plots, heatmaps, charts, and principal component analysis (PCA) to understand biological significance. (**H**) Pathway analysis was performed to integrate the lipidomic and cytokine datasets.

The objective of our study is to integrate exploratory lipidomic and cytokine profiles after mild traumatic brain injury (mTBI) to understand underlying biological processes. The experiments on experimental batch 1 were completed 1.5 years prior to experimental batch 2 (Figure 1A). A modified closed-head cortical control impact device was used to induce sham control, single mTBI (smTBI, 1X), and repetitive mTBI (rmTBI, 3X) injuries for both male and female rats (Figure 1B and Table S1). The 3X groups took a significantly longer time to right compared to the sham control. There were no differences between the sham and 1X groups (Table S1). The male and female 3X groups took significantly longer to right compared to their respective sham control counterparts. This result indicates acute neurological effects following repetitive injury for both combined and separate sexes. The 54 combined brain cortices from experimental batch 1 and 2 were harvested for lipidomic analysis by non-targeted ultra-high-performance liquid chromatography mass spectrometry (UPLC-MS). Fifty-three brain cortices were used for cytokine analysis by a Luminex multiplex assay (Figure 1C,D) due to a missing sample. We further processed the data by reducing features to minimize batch effects and obtain annotated features to investigate biological relevance of lipid and inflammation markers (Figure 1E–H).

## 4. Results

### 4.1. Brain Lipidomic Changes after mTBI

#### 4.1.1. Statistical Analysis of Lipid Content in Brain Cortices Detected by LC-MS

**Figure 2:**
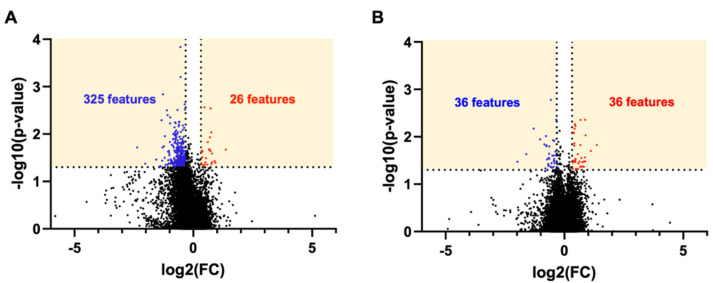
Volcano plot of all features detected by LC-MS. (**A**) 3X vs. sham control. (**B**) 1X vs. sham control. The numbers correspond to features that were both statistically significant *p* < 0.05 and had 1.25-fold change. Blue denotes decrease and red denotes increase. Statistical analysis: The *p*-values were calculated by ANOVA and TukeyHSD-post hoc by Compound Discoverer among all three groups (sham vs. 1X, sham vs. 3X, 1X vs. 3X). The *p*-values from these calculations were extracted for the volcano plots.

We evaluated the lipidomic profiles of brain tissue after mTBI. There were 22,735 features detected in the 54 cortices by UPLC-MS in the combined samples from experimental batches 1 and 2. The volcano plot analysis of the lipidomic dataset illustrates a relative fold change and *p*-values. Features in the yellow boxes denote an overlap greater than a |1.25|-fold change and statistically significant differences (*p* < 0.05) (Figure 2) between the injured and sham groups. The results indicate that 325 features decreased, and 26 features increased in the 3X injury group relative to the sham group (Figure 2A), which suggests lipid depletion or turnover due to injury. In the 1X group relative to the sham control group, 36 features decreased, and 36 features increased (Figure 2B). The results of the volcano plot illustrate greater changes due to injury in the 3X group compared to the 1X groups. Interestingly, there was a more significant decrease in lipids in the 3X injury group that may be a result of a different injury time course for the more severe injury condition.

#### 4.1.2. Minimization of Experimental Batch Effects in the Brain Cortices Dataset

**Figure 3:**
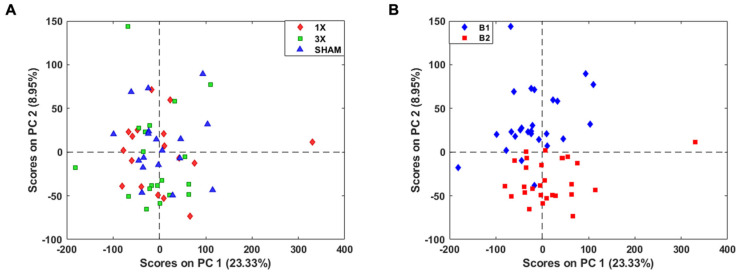
All 22,735 features detected by LC-MS show prominent batch effects. (**A**) PCA score plot of all features shows no separation between injury groups. (**B**) PCA score plot of features depict separation of experimental batches along PC2. B1 denotes batch 1 and B2 denotes batch 2.

We detected 22,735 features in the lipidomic dataset with combined experimental batches 1 and 2. A PCA score plot of all features indicated no separation between injury severity and sex (Figure 3A and Figure S1). Therefore, there was a need for additional feature reduction to explore altered lipid dysfunction due to injury. To further analyze the brain lipidome following mTBI, we evaluated experimental batch effects from the combined samples. A PCA score plot of all 22,735 features showed prominent separation along PC2 of experimental batch 1 and 2, likely primarily due to variable sample storage times (Figure 3B). There were no batch effects among the internal standards, which aided as a quality control metric confirming that batch effects were due to experimental effects. We expected tissue age to be a variable in our study.

**Figure 4:**
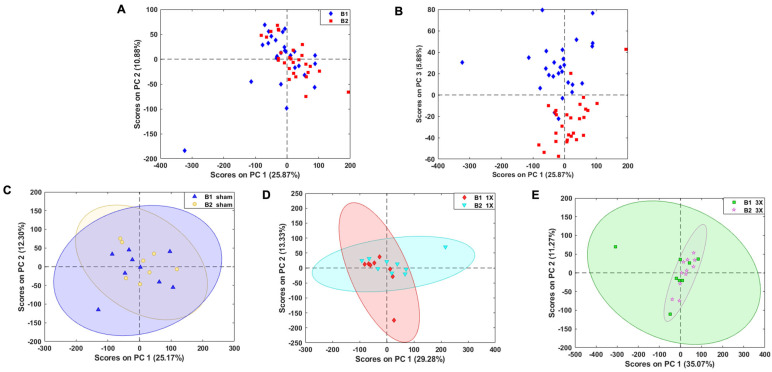
19,411 features remained after feature reduction of batch effects. (**A**) PCA score plot of reduced features depict negligible separation of batches along PC1 and PC2. (**B**) PCA score plot of 19,752 features show separation of batches along PC3. PCA score plot depicting overlap of confidence ellipses between (**C**) sham control batches (**D**) 1X batches and (**E**) 3X batches. B1 denotes batch 1 and B2 denotes batch 2.

The batch effect explained ~9% of the variance along PC2; therefore, we evaluated different methods to further minimize batch effects due to differences in storage time and conditions. We first calculated the *p*-values using Welch’s *t*-test on the complete dataset that was logarithmically transformed and found 14,600 features with no statistically significant differences between batch 1 and 2 (Figure S2). The PCA score plot indicated no separation between batches. However, with this method, we may have removed features with biological relevance to TBI. Therefore, we calculated the *p*-values of each feature using Welch’s t-test between sham control batches 1 and 2 on logarithmic transformed data to minimize batch effects without removing biologically significant molecules. After this comparison, 19,411 features remained that were not statistically significant (*p* > 0.05) between sham control batches 1 and 2. The PCA score plot depicted minimal batch effects along PC1 and PC2 (Figure 4A). However, there remained batch effects along PC 3, which explained ~6% of the variance (Figure 4B), which was deemed to be acceptable. This feature reduction method enabled us to minimize high inter-batch effects while potentially retaining a higher number of biologically relevant molecules. The PCA score plot depicts a prominent overlap of the samples and confidence ellipses between the sham (Figure 4C), 1X (Figure 4D), and 3X (Figure 4E) batches. The 3324 features removed depict prominent clustering of batch 1 and batch 2 (Figure S3) along PC1. These results allowed us to combine batch 1 and batch 2 samples for the analysis of brain lipidome changes after mTBI.

#### 4.1.3. Lipid Annotation

**Figure 5:**
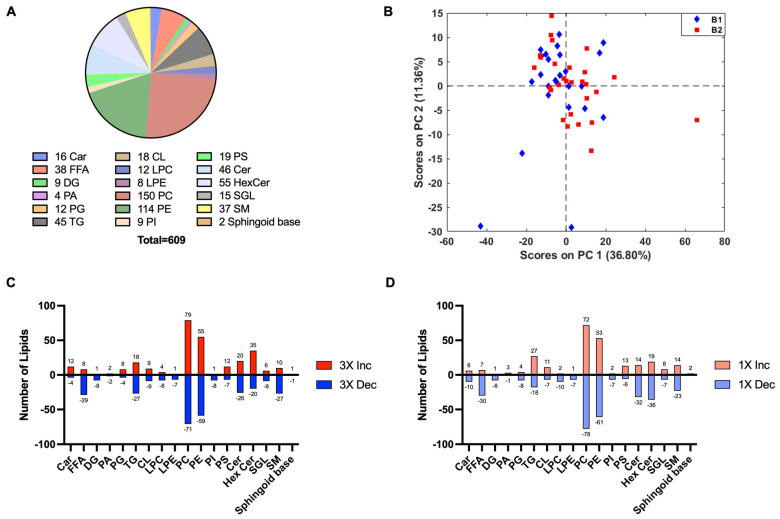
Annotated lipids in the lipidomic dataset. (**A**) Pie chart depicting the 609 annotated lipids belonging to each lipid subclass. Data are the named subset from the total features detected by LC-MS. (**B**) PCA score plot of annotated lipids depict negligible batch effects. (**C**) The 3X injury group relative to sham control. (**D**) The 1X injury group relative to sham control. Bar chart shows number of lipids that either increase (+) or decreased (−) due to TBI. Car—carnitine; FFA—free fatty acids; DG-diacylglycerols; PA—phosphatic acid; PG—phosphatidylglycerols; TG—triacylglycerols; CL—cardiolipin; LPC-lysophosphatidylcholine; LPE-lysophosphatidylethanolamine; PC—phosphatidylcholine; PE-phosphatidylethanolamine; PI-phosphatidylinositol; PS-phosphatidylserine; Cer—ceramide; HexCer—hexosylceramide; SGL—sulfoglycolipids; SM—sphingomyelin; sphingoid base.

The 19,411 features were matched against an in-house database to identify lipid subclasses and classes. There were 609 tentatively annotated lipids in the dataset at an MSI level 2 [43] (Figure 5). The isotopic features were summed [22] and resulting features were divided into subclasses. These included lipids from the carnitine (Car) and free fatty acids (FFAs) subclasses (fatty acid class), diacylglycerols (DG), phosphatic acid (PA), phosphatidylglycerols (PG), and triacylglycerols (TG) subclasses (glycerolipid class), cardiolipin (CL), lysophosphatidylcholine (LPC), lysophosphatidylethanolamine (LPE), phosphatidylcholine (PC), phosphatidylethanolamine (PE), phosphatidylinositol (PI), and phosphatidylserine (PS) (phospholipid class), and ceramides (Cer), hexosylceramides (HexCer), sulfoglycolipids (SGL), sphingomyelins (SM), and sphingoid bases (sphingolipid class) (Figure 5A). PCA analysis showed the annotated features that had no batch effects (Figure 5B). The bar chart depicts the number of lipids that either increased or decreased in the 3X injury group relative to the sham control group (Figure 5C). In total, 75% of the Car subclasses increased and 78% of the FFA and 73% of the SM decreased in the 3X injury group relative to the sham control group (Figure 5C). DG(16:0_22:4_0:0) PC(O-37:5)/PE(O-40:5)/PE(P-40:4), and PE(O-16:1/20:3) significantly increased in the 3X group relative to the sham control group. Additionally, tetradecanedioic acid, Arachidic acid, FA(22:2), FA(28:0), Myristic acid, Stearic acid, TG(36:2), TG(54:4), and TG(58:9) significantly decreased in the 3X injury group relative to the sham control group. In total, 63% of the Car and 81% of the FFA subclasses decreased in the 1X injury group relative to the sham control group (Figure 5D). PC(30:0) > PC(14:0_16:0), PC(38:2) > PC(18:1_20:1), PC(O-38:5), PE(O-32:1) > PE(O-16:1/16:0), and PS(O-40:7) > PS(O-20:3/20:4) significantly increased due to 1X injury relative to the sham control group. Carnitine, FA(18:3), linoleic acid, palmitoleic acid, trans-10-Heptadecenoic acid, tetradecanedioic acid, FA(23:0), FA(22:6), FA(17:0), FA(22:4), PC(40:8) > PC(20:4_PC(14:0_16:0), PE(O-16:1/16:0), and PS(O-20:3/20:4), PC(41:7), PE(36:2) > PE(18:1/18:1), PE(O-38:5) > PE(O-18:1/20:4), PE(42:10) > PE(20:4_22:6), PE(36:2) > PE(18:2_18:0), PE(O-40:7) > PE(O-18:2/22:5), PE(O-42:7) > PE(O-18:2/24:5), and PE(36:3) > PE(18:1_18:2) all significantly decreased in the 1X injury group relative to the sham control group.

### 4.2. Evaluation of Inflammation Markers after mTBI

**Figure 6:**
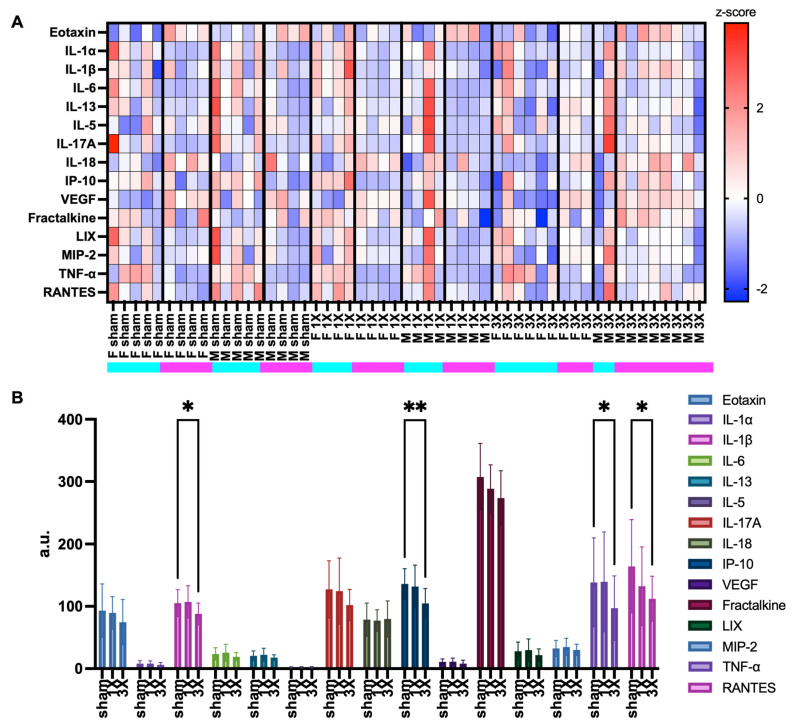
Chemokine and cytokine inflammation marker profiles. (**A**) Heatmap of cytokine and chemokine expression z-score values. Rows are z-scored. (**B**) Levels of chemokine and cytokine markers in the brain cortices. Graph denotes mean and standard error of mean (SEM). Statistical analysis, Mixed-effect analysis, and Tukey’s post hoc test. *, *p* < 0.05; **, *p* < 0.01.

Tissue samples from the same 53 brain cortices were used to evaluate a panel of inflammation markers: eotaxin, IL-1a, IL-1B, IL-6, IL-13, IL-5, IL-17a, IL-18, IP-10, VEGF, fractalkine, LIX, MIP-2, TNF-α, and RANTES (Figure 5). One sample from the 54 total samples was removed based on a technical error. Additionally, batch 1 experimental samples were completed and run at a separate time than batch 2. The z-scores for batch 1 and 2 independently depicted variability between batches (Figure S4). The heatmap z-scores were computed for each animal (Figure 6A). The heatmap suggested variability in cytokine expression in each group. The female 3X pro-inflammatory profile decreased due to repetitive injury, which may be due to the neuroprotective effects of female reproductive hormones [44,45,46]. We further evaluated the effect of injury on each cytokine. Surprisingly, the results indicated a significant decrease in the 3X injury group relative to the sham control group in IL-1B, IP-10, TNF-α, and RANTES (Figure 6B). Interestingly, there were also many lipids that significantly decreased in the original, 22,735 feature dataset (see Figure 2B). There was a significant decrease in IP-10 and VEGF in the 3X group relative to both the sham control and 1X injury groups between the batch 1 samples and a significant decrease in TNF-α in the 3X group compared to the sham group (Figure S5). There was a significant decrease in eotaxin in the 3X group relative to the sham control group in batch 2 (Figure S6). The data were further analyzed for sex differences in the inflammatory profiles after mTBI (Figure S7). The only significant difference between sex was with the expression of IL-18, which was higher in males compared to females.

### 4.3. Integration of Lipidomics and Cytokine Datasets

**Figure 7:**
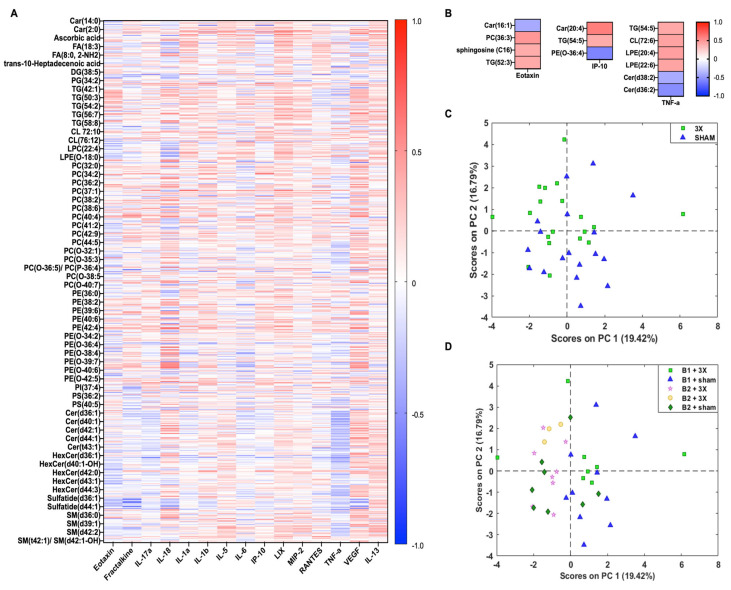
Correlation between annotated lipids and cytokines following 3xmTBI. (**A**) Spearman correlation heatmap of 611 annotated lipids and 15 cytokines. List based on subclass. (**B**) Spearman correlation heatmap of reduced lipids and cytokines that had a 1.25-FC in either direction between sham and 1X injury groups. (**C**) PCA score plot of 12 lipids depicting sham control and 3X injury groups. (**D**) PCA score plot of 12 lipids depicting experimental batches and injury severity.

The annotated lipids and inflammation markers were directly compared to better understand biological relevance following mTBI. The 609 annotated lipids from the 3X injury group were correlated with 15 cytokine panel of the 3X injury (Figure 7A). The data showed negative correlations with eotaxin, TNF-α, IP-10, and RANTES. We further filtered cytokines and lipids that had possible injury effects. The datasets were reduced with the following criteria: |1.25|-fold change and Spearman R values |0.4|, which left twelve annotated lipids and three cytokines (Figure 7B). The data showed no clear trends, except for the sphingolipid class, which negatively correlated with TNF-α. The lipids with high fold change values underwent PCA analysis to visualize injury effect. (Figure 7C,D). The results illustrate no separation between sham control and injured groups; there appears to be a slight shift in the 3X samples along PC2 (Figure 7C). Despite data processing of batch effects, there remains a separation between the experimental batch groups (Figure 7D).

**Figure 8:**
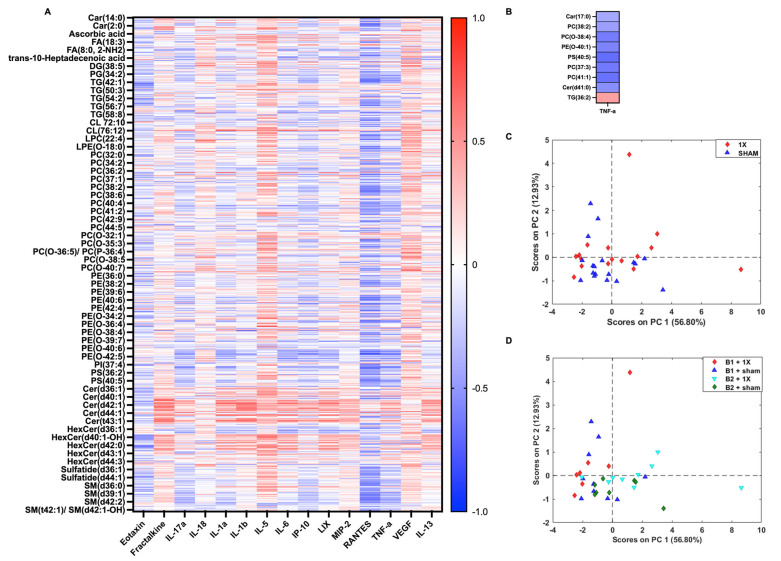
Correlation between annotated lipids and cytokines following 1xmTBI (**A**) Spearman correlation matrix of 52 annotated lipids and 1 cytokine. Heatmap denotes the median values of each injury group. (**B**) PCA score plot of lipids that depicts a slight shift along the diagonal of PC1 and PC2 between sham and 1X samples. (**C**) PCA score plot of 9 lipids depicting sham control and 3X injury groups. (**D**) PCA score plot of 9 lipids depicting experimental batches and injury severity.

The same analysis for lipid and cytokine comparison (see Figure 7) was completed to integrate the sham and 1X groups. The 609 annotated lipids from the 1X injury group were correlated with 15 cytokine panel from the 1X injury group (Figure 8A). The data showed negative correlations of eotaxin, TNF-α, IP-10, and RANTES with the lipids. There were nine lipids and one cytokine that had a fold change of |1.25| relative to sham control and a Spearman R value of |0.4| (Figure 8). The PCA score plot of the nine lipids with high fold changes between the sham and 1X injury groups depicted no clear separation between the sham control and 1X injury groups (Figure 8B). Similarly, to the 3X samples, there was a prominent batch effect.

## 5. Discussion

### 5.1. Combining Lipidomic and Cytokine Data

Brain lipid and cytokine profiles were integrated after single and repetitive mTBIs in male and female rats in the acute injury phase following a closed-head injury. UPLC-MS and a Luminex multiplex cytokine assay were used to measure lipids and cytokines, respectively. The experiments on experimental batches 1 and 2 were completed at different times which led to differences in sample age, baseline differences in animals, tissue storage time, and technical variability in inducing injury. The combined datasets were processed to minimize batch effects while removing the least amount of biologically relevant molecules. The processed lipidomic dataset was annotated with an in-house database and the features were further reduced to identify changes due to injury. Brain samples from the same cortical tissue were also processed to evaluate inflammatory responses due to injury. The work in this manuscript illustrates the integration of data from non-targeted lipidomics and an assay for selected pro- and anti-inflammatory markers after (1X) smTBI and (3X) rmTBI, a complex and dynamic neural disorder.

A major finding from the study is the identification of a set of annotated lipids that decreased due to injury in our repetitive mTBI model. There were 325 lipid and small metabolite features that had high fold changes and statistically significant decreases due to rmTBI and 36 features after smTBI. Most of the free fatty acids, diacylglycerols, and sphingomyelin subclasses decreased in the cortex in both the single and repetitive mTBI groups. This result is inconsistent with other studies that found an increase in free fatty acids, diacylglycerols, and sphingomyelin 24 h post-injury [47,48,49], which may be due to the differences in our closed-head injury model versus the open-head model in other studies. A closed-head injury produces a diffuse injury, which may lead to a different injury cascade time course compared to direct brain impacts. Further studies and sophisticated analyses are needed to elucidate global lipidome alterations between different TBI injury models for comparisons between studies. Alternatively, there could be different changes in various brain regions that are more prone to damage. Follow-up lipidomic studies should investigate regional differences, as well as temporal changes.

Another major result of our study is that proinflammatory cytokines, IL-1B, IP-10, TNF-α, and RANTES, surprisingly decreased after rmTBI. There were no statistical differences between smTBI and sham control cytokine profiles. Although the rmTBI animals took significantly longer to right immediately following the impacts compared to sham control, there was a decrease in proinflammatory cytokines 24 h post injury. This may be due to biphasic or multi-phasic neuroinflammation cycles in the brain after injury. A study that investigated cytokine temporal profiles in the mouse cortex after a single TBI in the acute, subacute, and chronic phases found a significant increase in VEGF-B across all time points from 8 h to 30 days relative to a sham group, but VEGF-B was lowest at 24 h and 30 days [50]. The same study also showed a decrease in fractalkine 24, 48, 72 h, 7 and 14 days and a decrease in eotaxin levels at 24 h and RANTES at 72, 96 h, and 7 days. Interestingly, IL-1β decreased 24 and 72 h after injury, which is consistent with our results in the rmTBI groups. It is also possible that the depletion of some lipids (e.g., PUFAs) that are necessary substrates for inflammatory mediators causes a downstream reduction in cytokines [51].

Another interesting finding from our study is that some lipids that were correlated with cytokine expression showed an injury effect, albeit a minor one. We detected changes in carnitine and free fatty acid subclasses. In our smTBI model, we found most of the species in the carnitine subclass decreased, whereas the subclass increased in our rmTBI model. Carnitines are a significant contributor to fatty acid metabolism in the brain [52], play a role in neurotransmission [53], and support neuroprotection [52]. Previous clinical and experimental studies have shown that acylcarnitine supplementation improved memory and cognition in Alzheimer’s disease [54], reduced cell death in primary neuronal cells [55], and improved balance and reduced overall lesion volume after a TBI [56]. In the present study, we show a moderate negative correlation between Car(16:1) and eotaxin, where Car(16:1) increased and eotaxin decreased due to rmTBI. However, Car(17:0) negatively correlated with TNF-α in the smTBI group, where Car(17:0) decreased and TNF-α increased relative to the sham control group. These results suggest that the carnitine-containing lipids may provide neuroprotective effects after TBI, warranting further investigation. Free fatty acids in the brain are predominantly obtained through diet via the blood [57]. We found that there were no free fatty acid changes that correlated with cytokine changes in either the single or repetitive mTBI model. However, the majority of the free fatty acid subclass decreased due to TBI after both single and repetitive mTBI. A previous study found an accumulation of FFA and DGs in the injured sensorimotor cortex after TBI at 30-min and 24-h [47]. Another study found a significant elevation of FFA(16:0), FFA(18:0), FFA(18:1), and arachidonic acid in the injury site at 30 min, 2.5 and 24 h after CCI [58]. These mixed results suggest the need for further exploration of global lipidome changes after a closed-head mTBI.

Glycerolipids are important in intracellular signaling processes [59] and membrane formation [60]. The glycerolipids we detected in our dataset were DG, PA, TG, GPL, and PG. DGs are a potential source for some endocannabinoids and disturb the biosynthetic pathway which is implicated in neurogenesis and synaptic plasticity [61]. In our study, the DG and TG subclasses decreased due to single and repetitive mTBI. This result is consistent with the findings from a matrix-assisted laser desorption/ionization (MALDI) imaging study using an open-head CCI model, which showed a decrease in DAG(40a:6) at 1 day post injury but showed a marked increase at 3 days [48]. However, another study showed a marked increase in DGs in the acute phase after injury [58]. Although there were no DGs that reached the fold change criterion, we found that DG(34:1) and DG(40:6) negatively correlated with TNF-α and DG(36:1) positively correlated with TNF-α in the smTBI group. TNF-α and IL-1 upregulation from glutamate excitotoxicity activates PC-phospholipase C (PC-PLC) which hydrolyzes PC, to release DG and phosphocholine. Therefore, we expected to see a positive correlation between DGs and inflammatory markers after TBI. We found that TG(52:3) positively correlated with eotaxin, and TG(54:5) positively correlated with IP-10 and TNF-α. Triglycerides have been shown to cross the blood–brain barrier (BBB) and induce insulin resistance [62] and break down during autophagy [63,64]. A study found exogenous TGs induced an increase in TNF-α expression in Jukart T cells [65]. Further studies are needed to probe pathways to understand glycerolipid changes after TBI.

Phospholipids are an important constituent of cellular membranes by providing structural support [66]. They also function in intracellular signaling and facilitating inflammatory responses through the activation of excitotoxicity and mitochondrial dysfunction [11,67]. We detected different subclasses under the phospholipid class, such as CL, LPC, LPE, PC, PE, PI, and PS. We found positive correlations between PC(36:3) and eotaxin, and CL(72:6), LPE(20:4), and LPE(22:6) with TNF-α in the rmTBI group. There was a negative correlation between PE(O-36:4) and IP-10 in the rmTBI group. For the smTBI group there were negative correlations between PC(38:2), PC(O-38:4), PE(O-40:1), PS(40:5), PC(37:3), and PC(41:1) with TNF-α. PCs are hydrolyzed by phospholipases A2, PC-LPC, and phospholipase D(PLD), which releases lipid byproducts such as arachidonic acid (AA) and docosahexaenoic acid (DHA) from the membrane, lipids that have both pro- and anti-inflammatory effects [11,68]. CL are phospholipids that are found in the inner mitochondrial layers and play an important role in bioenergetics and signaling [69,70,71]. There was no clear trend for our CL species where 50% of the molecules either increased or decreased in the rmTBI group relative to the control, and 39% decreased and the other 61% increased due to smTBI. This result suggests a graded injury difference between the smTBI and rmTBI groups. CL(72:6) positively correlated with TNf-α in the rmTBI but no correlation was found in the smTBI groups. A previous preclinical, pediatric TBI study found a decrease in CL in the injured site at 4- and 24-h after injury [71]. Phospholipids play an important role in metabolism and inflammation after a TBI [68], therefore further studies investigating the role of individual phospholipid species are warranted.

Sphingolipids are enriched in the brain and essential for the central nervous system structural integrity, as well as signal transduction and tissue development [72,73]. We detected Cer, HexCer, SM, sphingoid bases, and SGL in our lipidomic dataset. We found a positive correlation between sphingosine(C16) and eotaxin and a negative correlation between Cer(d36:2) and Cer(d38:2) and TNF-α in the rmTBI group. There was a positive correlation between Cer(41:0) and TNF-α in the smTBI group. Sphingosine is a bioactive sphingolipid that is shown to play a role in apoptosis [74]. Sphingosine has been shown to be elevated after a TBI and 1, 2, and 7 days after open-head TBI, and the role of sphingolipid in mitochondrial dysfunction has also been shown [75]. In our study, we found that sphingosine increased due to TBI, which is consistent with other studies. Ceramides and sphingomyelins have been shown to increase in the injured site at 24 and 72 h post TBI [48]. The upregulation of TNF-α and IL-1 after a stroke activates phospholipases and sphingomyelinase, which results in the loss of PC and SM but the elevation of AA and Cer [11]. However, we find both increases and decreases in Cer and SM after TBI. Further studies are needed to elucidate the role of novel markers after TBI.

### 5.2. Limitations, Challenges, and Opportunities

In this study, we integrated lipids and cytokines to further understand changes due to TBI. The study includes a large sample size and both sexes. In this section, we discuss the limitations of the presented work and current challenges and opportunities in the field of mass spectrometry for lipidomics. A semi-quantitative, untargeted mass spectrometry approach was used to analyze lipid alterations after mTBI in a clinically relevant animal model to maximize the detection of lipids in each sample [76,77,78]. This non-targeted technique allowed for a more unbiased analysis method for exploratory lipidomics compared to targeted approaches, which measure ions for predefined known lipids [79]. Yet, current non-targeted metabolomic techniques may not be truly unbiased due to the lack of detection of the complete lipidome in the sample [80]. A limitation of our study is that only approximately ~3% of the dataset was explored due to only obtaining a small subset of named lipids. Challenges in lipid annotation limit the scope of the lipid class and subclass alterations that can be detected after TBI. This, in turn, may lead to biased conclusions in exploratory research for diagnostic and prognostic TBI biomarkers.

A challenge in the field of lipidomics is the issue of replicability between studies, both between and within laboratories. Our current study design presented a challenge due to storage batch effects, sample processing variance, and LC-MS sensitivity drift. A study investigated lipidome storage conditions between tissues and found no significant degradation in the spectral data between 24 h and 7 months using desorption electrospray ionization (DESI)-MS [81], but deleterious effects with freeze/thaw cycles [81,82]. In our experiments, we saw prominent batch effects even between sham animals. We attempted to control for time under anesthesia and time of day for injuries to control for circadian rhythms. The batch effect in our study may be tied to both biological and procedural differences between the batches. To overcome batch effects, we removed features that were statistically different between batch 1 and batch 2. However, there were batch effects present along PC2 for the 12 lipids that correlated with some of the proinflammatory cytokines. This suggests that further methods are needed to minimize variance within a reduced lipid panel. An opportunity to overcome these barriers is to explore machine learning techniques such as Systematical Error Removal using Random Forest (SERRF) [83], ComBat [84], Harman [85], etc. This will aid in the translation of biomarkers from preclinical to clinical TBI studies.

Multi-omic TBI studies provide the ability to relate epigenomic, metabolomic, lipidomic, proteomic, and transcriptomics for biological interpretation of pathology. This can lead to more unbiased, hypothesis-driven questions and provide potential biomarker targets. We integrated multidimensional lipidomic data with a targeted cytokine panel, which added complexity because our non-targeted technique did not capture many downstream low abundant oxidized lipids, which are known to be involved in inflammatory pathways [68].

Lastly, a major hurdle in the field is relating lipidomic changes to their biological relevance in TBI. Lipids are not genetically encoded; therefore, examining the function of individual lipids remains a major challenge [86]. Current methods to probe biological significance in the field of lipidomics are through pathway analysis aids such as MetaboAnalyst [87], LIPID MAPS [88], LipidSig [89], and LIPEA [90], to name a few, to tie lipids to known metabolic pathways. In this study, we sought to undertake a multi-omics approach by correlating a small fraction of known lipids to cytokines that align with neuroinflammation in order to draw conclusions of biological significance. Most drugs target proteins; therefore, the integration of various domains such as epigenetics, metabolomics, lipidomics, and epilipidomics lends additional dimensions that may ultimately provide novel and effective targets. This integration provides an opportunity to link known and well-understood cytokine changes to lipidomic changes to better understand TBI metabolic cascades. In our present work, there were moderate correlations between some lipids and cytokines with modest fold changes down or up. Yet, there remained the challenge of acquiring biological significance due to our use of a mild TBI model and the analysis of one time point after injury. Closed-head mild TBI models usually lack gross tissue damage and produce a diffuse injury that may lead to different cellular damage in various regions of the brain [23]. For this study, we chose to examine the frontoparietal cortical tissue directly under the impact site. Other brain regions are likely affected, and an examination of multiple regions would lead to an increased understanding of the spatial aspects of TBI. Previous studies have shown that PC, SM, and PE decreased in the cortices but increased in the hippocampi 3 months post-open head CCI [67]. There may be disparate lipidomic signatures in different brain regions after single and repetitive mTBI. Another limitation of our current study is we investigated lipidome and cytokine changes 24 h after injury. This is only a single snapshot of the metabolic processes within the acute injury phase. A study used a weight drop TBI model and found a 2-fold increase in LPC at 4 h and a 5-fold increase at 24 h using a pediatric model of TBI [71]. They also found a decrease in CL at both 4 and 24 h after injury. Another study found no significant changes in PCs at 2, 4, 6, 24, 48, and 120 h post TBI [91]. There is a need for further analysis of brain lipidome and cytokine temporal profiles to understand biological significance using larger sample sizes and robust machine learning tools.

## 6. Conclusions

Cumulatively, we present lipidome changes after single and repetitive mTBI that correlate with cytokines to understand biological relevance. This study presents some challenges and opportunities in the field of TBI and mass spectrometry to understand the biological significance of lipidome changes in light of neuroinflammatory changes after a TBI. As more robust analysis tools become available, novel hypotheses can be tested that utilize the combination of omics approaches with assays that target known molecules. The balance between lipid metabolism and neuroinflammation is the basis for anti-inflammatory and pro-resolving bioactive lipid pharmacologic agents and even diet supplementation [92]. A framework of using known pathophysiologic changes such as those identified in the present study can reveal signaling pathways and advance identification of novel therapeutic targets.

## Data Availability

Data are available through the NIH Metabolomics Workbench [93] supported by NIH grant U2C-DK119886 and OT2-OD030544 under study ID ST003093 (http://dx.doi.org/10.21228/M88T6X (released date on 21 March 2024)). Raw data are provided RAW format, data processed with Compound Discoverer is available in an excel file.

## Supplementary Materials

The following supporting information can be downloaded at: https://www.mdpi.com/article/10.3390/metabo14030133/s1, Figure S1: 22,735 features detected by LC-MS; Figure S2: Feature reduction to minimize batch effects with full dataset; Figure S3: 2983 features with statistically significant difference between batches; Figure S4: Heatmap of cytokine and chemokine expression z-scores; Figure S5: Inflammation marker profiles in batch 1; Figure S6: Inflammation marker profiles in batch 2; Figure S7: Inflammation marker profiles in female and male groups; Table S1: Experimental sample number and righting latency.
