# Supplementary material for: Integrative Analysis of Cytokine and Lipidomics Datasets Following Mild Traumatic Brain Injury in Rats
Source: Metabolites. 2024 Feb 21;14(3):133. doi: 10.3390/metabo14030133 (PMC10972386; doi:10.3390/metabo14030133)
Supplement: Supplementary file 1 [file metabolites-14-00133-s001.zip › metabolites-2865155-supplementary.pdf]

Supplementary Information

Integrative Analysis of Cytokine and Lipidomics Datasets Following Mild Traumatic Brain Injury in Rats

Alexis N. Pulliam [1,2], Alyssa F. Pybus [1,2], David A. Gaul [2,3], Samuel G. Moore [2,3], Levi B. Wood [1,2,4], Facundo M. Fernández [2,3] and Michelle C. LaPlaca [1,3,*]

[1] Coulter Department of Biomedical Engineering, Georgia Institute of Technology/Emory University, Atlanta, GA 30332, USA; afpybus@gatech.edu (A.F.P.); levi.wood@me.gatech.edu (L.B.W.)
[2] Petit Institute for Bioengineering and Bioscience, Georgia Institute of Technology, Atlanta, GA 30332, USA
[3] School of Chemistry and Biochemistry, Georgia Institute of Technology, Atlanta, GA 30332, USA
[4] George W. Woodruff School of Mechanical Engineering, Georgia Institute of Technology, Atlanta, GA 30332, USA
* Correspondence: michelle.laplaca@bme.gatech.edu

Table S1. Experimental sample numbers and righting latency

Injury Group	Male	Female	Combined	Comparisons
Sham control	N= 9	N= 9	N= 18	
				[2]sham: male vs. female; p=0.8102
Righting latency (min) (mean ± SEM)	2.536 ± 0.339	2.224 ± 0.477	2.384 ± 0.276	
1X	N= 8	N= 9	N= 17	[1]Sham vs. 1X; p=0.7206
				[2]Male: sham vs. 1X; p=0.8415
Righting latency (min) (mean ± SEM)	3.281 ± 0.353	2.890 ± 0.352	3.074 ± 0.247	[2]Female: sham vs. 1X; p= 0.8564<br>[2]1X: male vs. female; p=0.7631
3X	N= 10	N= 9	N= 19	[1]Sham vs. 3X****; p<0.0001
				[2]Male: sham vs. 3X***; p=0.0007
Righting latency (min) (mean ± SEM)	7.427± 1.132	5.543± 1.097	6.401 ± 0.795	[2]Female: sham vs. 3X*; p= 0.0157<br>[2]3X: male vs. female; p=0.0895
	N=27	N= 27	N= 54	

Sample number and righting latency following mild TBI. [1] One-way ANOVA, Tukey's post-hoc multiple comparisons test. [2]Two-way ANOVA, Tukey's post-hoc multiple comparisons test. *, P<0.05, **P<0.01, ***,P<0.001, ****,P<0.0001

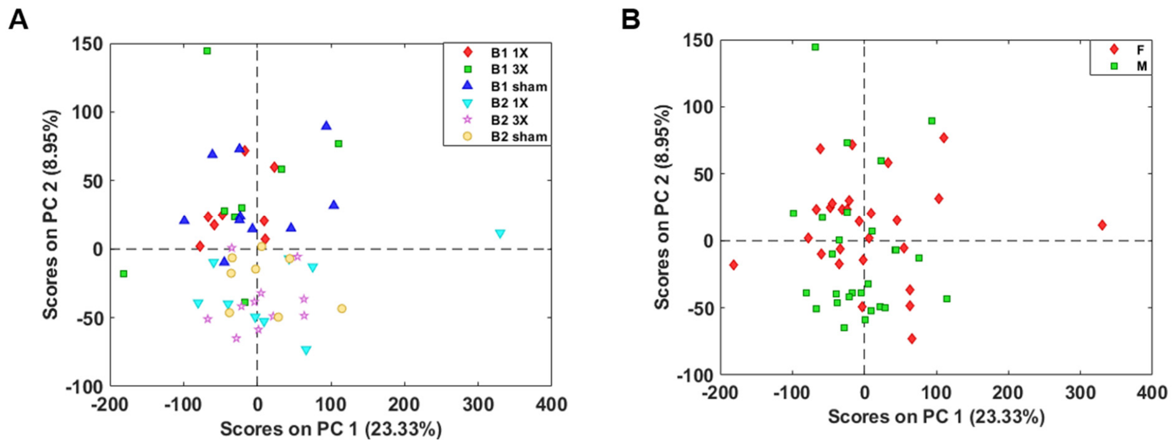

Figure S1. 22,735 features detected by LC-MS (**A**) PCA score plot depicts separation of batch 1 and batch 2 along PC2. Batch 1 samples are in upper quadrants. Batch 2 samples are in lower quadrants. (**B**) PCA score plot of all features indicate minimal separation between male and female samples.

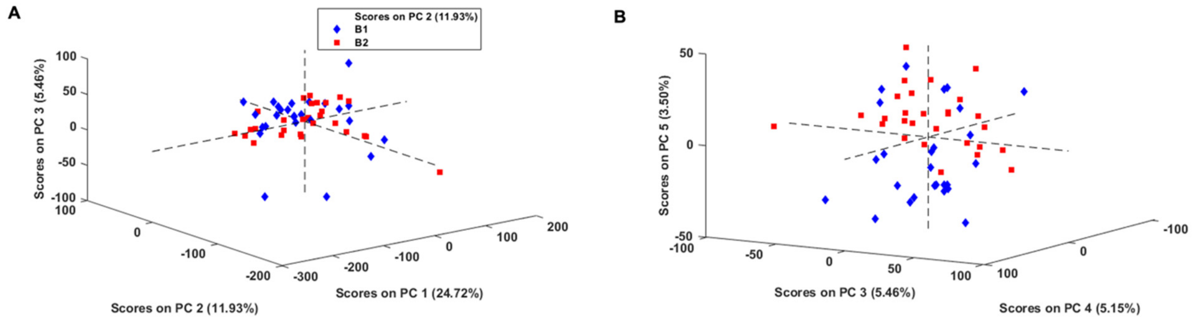

Figure S2. Feature reduction to minimize batch effects with full dataset (**A**) 3D-PCA score plot depicts no separation of batch 1 and batch 2 along PC1, 2, and 3. (**B**) 3D-PCA score plot of all features indicate separation of of batch 1 and 2 along PC5.

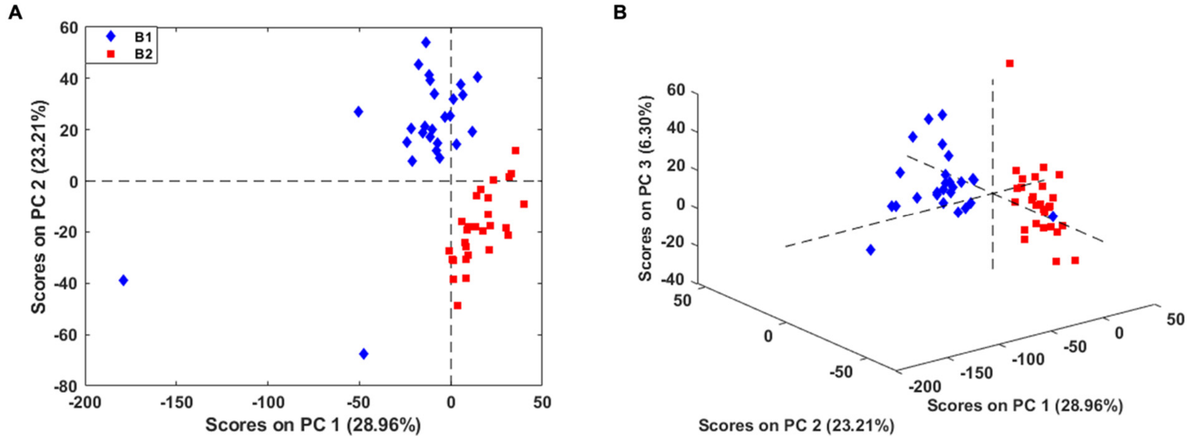

Figure S3. 2,983 features with statistically significant difference between batches. (A) 2-D PCA score plot show prominent separation of experimental batch 1 and 2 along PC 1 **(B)** 3-D PCA score plot show separation along PC1 and PC2. B1 denotes batch 1 and B2 denotes batch 2.

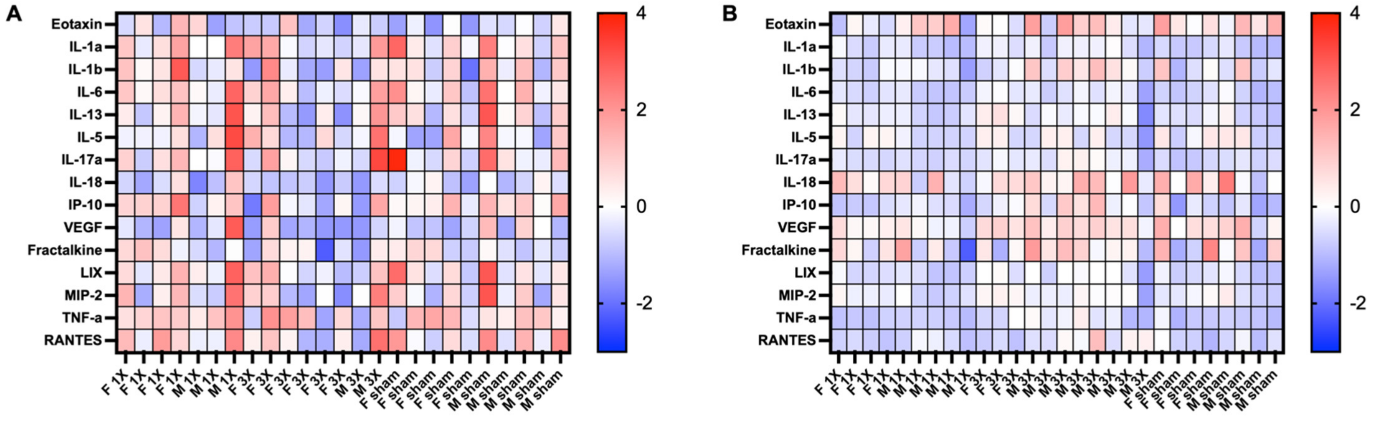

Figure S4. Heatmap of cytokine and chemokine expression z-scores. (**A**) batch 1 (**B**) batch 2.

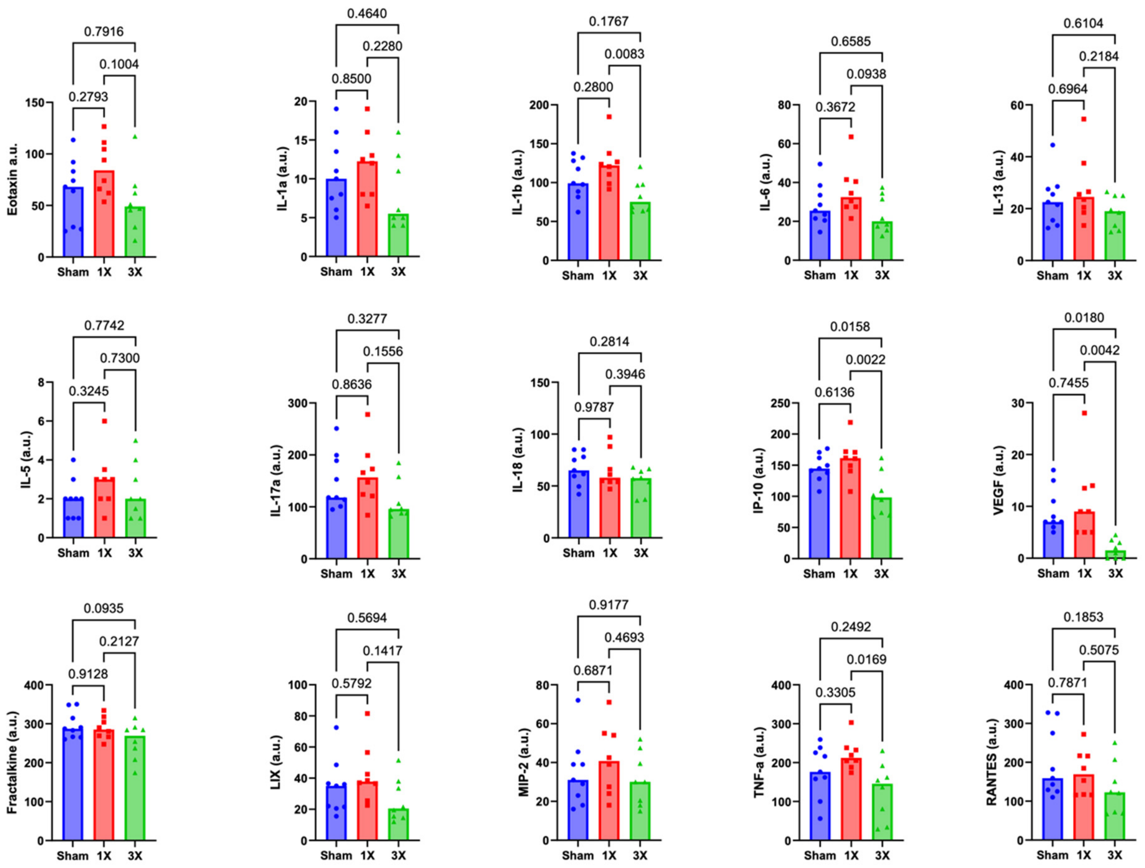

Figure S5. Inflammation marker profiles in batch 1. Data depict average cytokine and chemokine levels for each group. One-way ANOVA with Tukey's post-hoc, graph denotes mean.

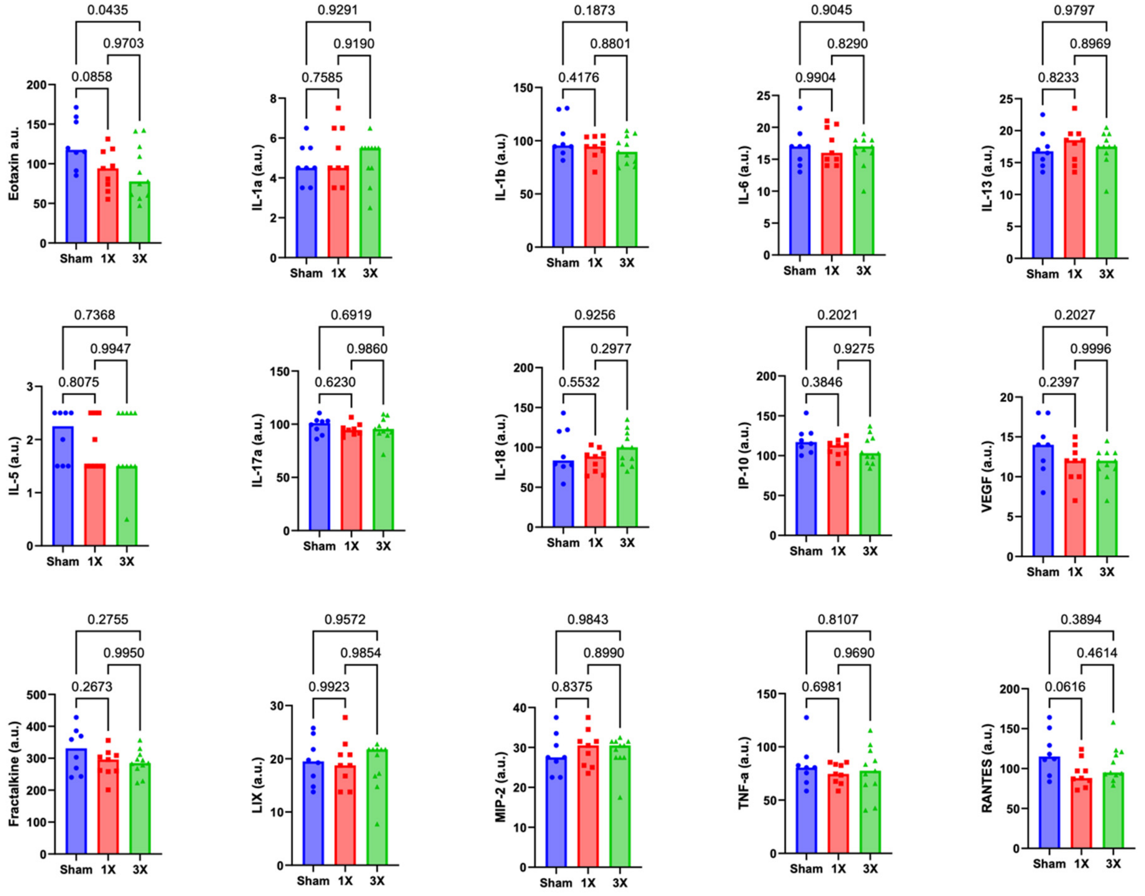

Figure S6. Inflammation marker profiles in batch 2. Data depict average cytokine and chemokine levels for each group. One-way ANOVA with Tukey's post-hoc, graph denotes mean.

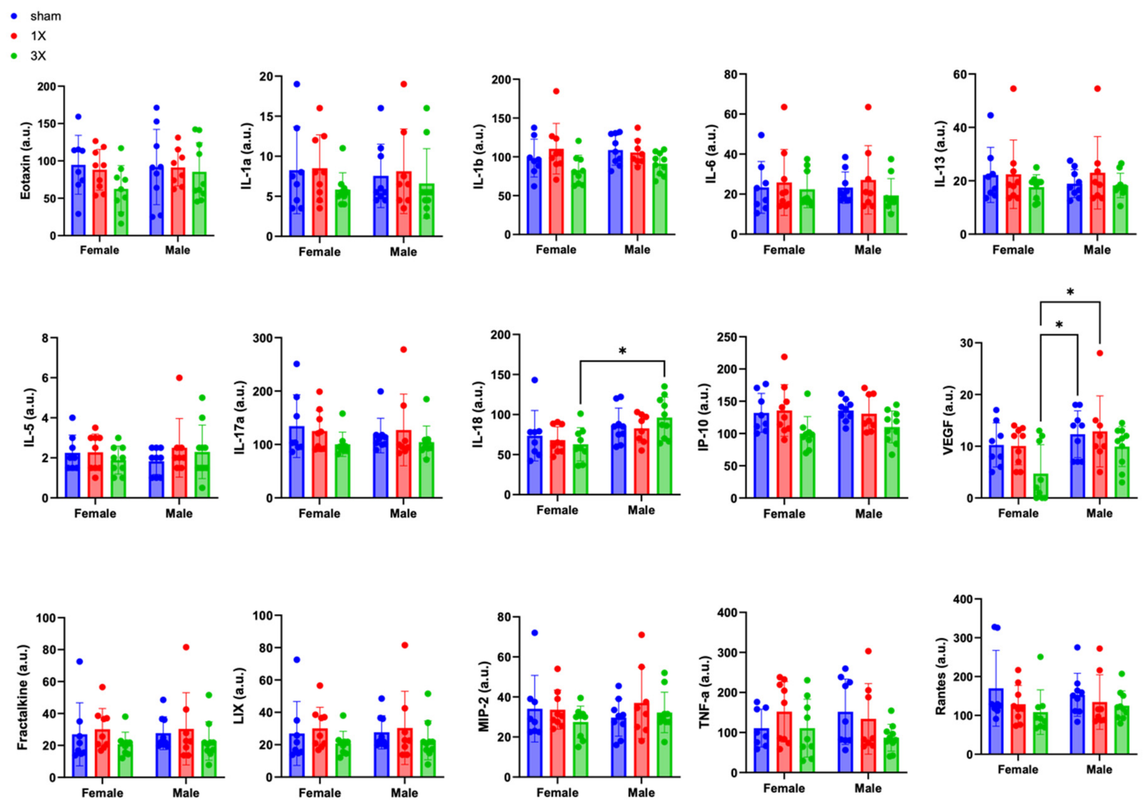

Figure S7. Inflammation marker profiles in female and male groups. Data depict average cytokine and chemokine levels for each injury group and sex. Two-way ANOVA with Tukey’s post-hoc, graph denotes mean.
